# Ethanol *versus* Phytochemicals in Wine: Oral Cancer Risk in a Light Drinking Perspective

**DOI:** 10.3390/ijms160817029

**Published:** 2015-07-27

**Authors:** Elena M. Varoni, Giovanni Lodi, Marcello Iriti

**Affiliations:** 1 Dipartimento di Scienze Biomediche, Chirurgiche ed Odontoiatriche, Università degli Studi di Milano, via Beldiletto 1/3, 20142 Milan, Italy; E-Mail: giovanni.lodi@unimi.it; 2 Dipartimento di Scienze Agrarie e Ambientali, Università degli Studi di Milano, via G. Celoria 2, 20122 Milan, Italy

**Keywords:** polyphenols, alcohol, risk factors, nutrition, oral squamous cell carcinoma, Mediterranean diet

## Abstract

This narrative review aims to summarize the current controversy on the balance between ethanol and phytochemicals in wine, focusing on light drinking and oral cancer. Extensive literature search included PUBMED and EMBASE databases to identify in human studies and systematic reviews (up to March 2015), which contributed to elucidate this issue. Independently from the type of beverage, meta-analyses considering light drinking (≤1 drinks/day or ≤12.5 g/day of ethanol) reported relative risks (RR) for oral, oro-pharyngeal, or upper aero-digestive tract cancers, ranging from 1.0 to 1.3. One meta-analysis measured the overall wine-specific RR, which corresponded to 2.1. Although little evidence exists on light wine intake, phytochemicals seem not to affect oral cancer risk, being probably present below the effective dosages and/or due to their low bioavailability. As expected, the risk of oral cancer, even in light drinking conditions, increases when associated with smoking habit and high-risk genotypes of alcohol and aldehyde dehydrogenases.

## 1. Introduction

In 2012, oral squamous cell carcinoma (OSCC) ranked as the 12th most common malignancy in Europe, affecting more than 73,000 European citizens of both sexes [1,2]. On what concerns mortality, stage of disease at diagnosis largely affects prognosis, with a five-year survival rate of 20% for advanced cases, corresponding to about 50% of OSCCs worldwide [3]. Prevention represents a major issue which is, currently, focused on avoiding tobacco smoking and reducing alcohol intake, the two most well-known risk factors for oral cancer [4].

In general, ethanol consumption, independently from the type of beverage, contemplates about 5% of all cancers, mainly of the liver [5], upper digestive and gastroenteric tracts, pancreas, breast, and lung [6,7,8,9,10,11]. In 2011, Western Europe had the 30% of oral cavity and pharynx cancers attributable to alcohol drinking [12], which raised to 44% for the upper aerodigestive tract cancers, *i.e.*, localized at oral cavity, pharynx, larynx, and esophagus [13]. Oral cancer has been, thus, causally associated with ethanol intake, particularly when consumed above the recommended upper limits of two drinks a day (30 g of ethanol/day) in men, and one drink a day (15 g of ethanol/day) in women [13,14]. These findings are indirectly supported by investigations on the role of alcohol-related cirrhosis not only as risk factor for hepatic cancer [5], but also for oral cancer, mainly in patients with potentially malignant oral disorders [15]. The increasing rate of liver cirrhosis, considered a surrogate marker of heavy ethanol intake, corresponded to growing incidence of oral cancer and related mortality [16]. In 2014, the International Agency for Research on Cancer (IARC) published a monograph on the carcinogenic effect of ethanol in humans [17], which confirmed these previous epidemiological studies [12,13,14].

Although evidences demonstrated heavy drinking is a major risk factor for oral cancer [10], the effect of light wine consumption on the oral carcinogenesis is still under debate, taking into account the topical activity, towards the oral mucosa cells, of the two xenobiotics present in wine, *i.e.*, ethanol and phytochemicals. Considering only the in-human studies, this review aims to analyze the putative roles of these wine components on oral cancer risk, focusing on the light drinking. Nonetheless, this survey also goes into moderate and heavy alcohol drinking as well, for comparison and for supporting the dose-dependent risk of disease and mortality. The authors independently carried out a comprehensive literature search on PUBMED and EMBASE databases, in order to identify systematic reviews and clinical studies on these issues, published in English, up to March 2015.

## 2. Wine Drinking and General Health: A Brief Overview

**Figure 1:**
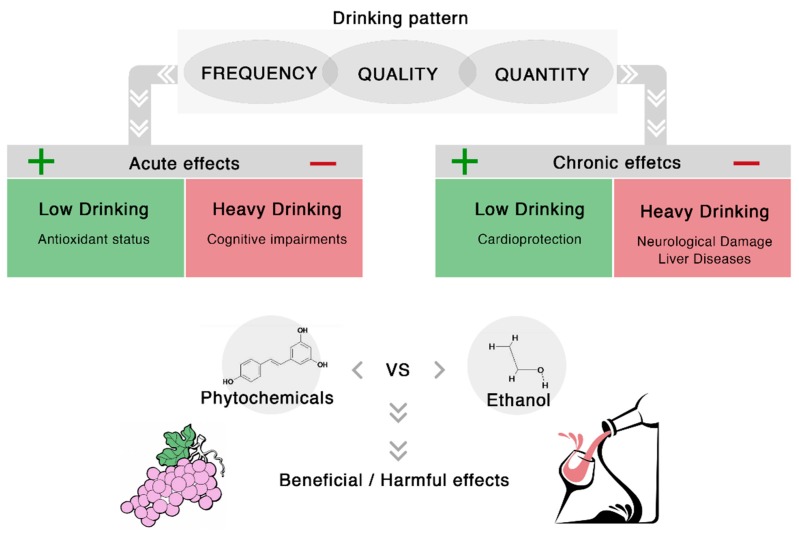
Model of alcohol consumption and health outcomes.

From the French paradox to date, a rising body of evidences reported health-promoting effects related to the habitual, low to moderate red wine intake at main meals [6,18]. Systematic reviews and meta-analyses supported, tough not unquestionably, that this nutritional habit may be protective against certain cardiovascular diseases, reducing morbidity and mortality rates [19,20]. The rationale is based on the high content of a variety of beneficial bioactive compounds, namely phytochemicals, contained in wine [21]. On other hand, the heavy wine intake (at least more than three drinks/day) has been strongly associated with a dose-dependent risk of liver diseases, such as fatty liver, cirrhosis and hepatic carcinoma, certain types of cancer, and brain damage, mainly cognitive function impairment [5,22,23] (Figure 1). Therefore, two cornerstones should be taken into account in patient risk analysis: (i) the individual drinking pattern; and (ii) the dietary habits.

### 2.1. Drinking Pattern

The drinking pattern corresponds to the different “dimensions” of subject drinking and includes the type of beverage, as well as how much, when and how often an alcoholic beverage is consumed [24]. Three main drinking scenarios can be identified, apart from alcoholism disorders, *i.e.*, abstention, low to moderate intake and heavy episodic drinking, the latter also called “binge” pattern. According to World Health Organization (WHO), the abstention group includes both “lifetime abstainers”, who have never consumed alcohol, and “former drinkers”, who previously consumed alcohol, but who did not in the last 12 months [25]. The heavy binge drinking group comprises mainly young subjects with an intake of at least 60 or more grams of pure ethanol (>5 drinks) on, at least, one occasion in the past seven days, generally during the weekend. In the middle, a regular and low to moderate consumption can be advocated: light alcohol intake, usually, corresponds up to one drink/day [26], while moderate consumption to one to two drinks/day [10]. The drinking pattern strongly affects health outcomes, at both short-term (acute) and long-term (chronic) level, with beneficial or harmful effects (Figure 1). Therefore, following a concept previously introduced for cardioprotection [21,27], the importance of drinking patterns was highlighted for oral cancer: in their epidemiological study, Petty and Scully suggested spirit drinkers usually consume high amount of alcohol in a unique occasion, with unbalanced high concentrations of ethanol over cancer-preventing phytochemicals [28]. Accordingly, the maximum oral cancer incidence and mortality are recorded in populations distributed in Eastern European countries, *i.e.*, Hungary, Slovakia, and Romania, where the consumption of spirits is higher than other nations [2] (Table 1). Similarly, in Italian North-Eastern regions, where the daily intake of spirits, in addition to wine, is a usual habit due to cultural traditions, the Italian highest percentage of oral cancer morbidity and mortality can be here observed [29]. These findings are also consistent with a recent study on Korean population, associating frequency of heavy binge drinking and mortality from oro-pharynx cancer [30]. However, further considerations on the association between individual alcohol abuse and general unhealthy behaviors are pivotal. Many reports suggest (un)healthy lifestyles tend to occur together, in particular the heavy binge alcohol drinking is associated with smoking habit, as well as to lower compliance to screening health programs [30].

**Table 1:** Recorded adult (15+) alcohol consumption by type of alcoholic beverage (in % of pure alcohol) in selected European and Mediterranean countries in 2005. Source: “European Status Report on Alcohol and Health”, WHO (World Health Organization) 2011 [31].

Country	Spirits (%)	Beer (%)	Wine (%)	Other ^1^ (%)
Italy	5	22	73	0
France	20	17	62	1
Portugal	10	31	55	4
Switzerland	18	31	50	1
Greece	26	24	49	1
Slovenia	13	39	48	0
Sweden	17	39	44	0
**Hungary** ^2^	**24**	**35**	**40**	**1**
Denmark	16	45	39	0
Morocco	13	50	37	0
Belgium	6	57	37	<1
Spain	13	45	36	6
Tunisia	5	63	32	0
United Kingdom	21	43	30	6
Norway	20	47	31	2
Germany	20	53	27	0
**Romania** ^2^	**39**	**39**	**22**	**0**
**Slovakia** ^2^	**49**	**36**	**15**	**0**
Egypt	33	56	11	0
Ukraine	61	32	7	<1
Estonia	57	34	7	2
Turkey	35	60	5	0
Russian Federation	63	33	1	3

^1^ Local traditional alcoholic beverages not included in the previous categories; ^2^ the top three European countries for the highest incidence and mortality rates of lip, oral cavity, and pharynx cancers are highlighted in bold.

### 2.2. Dietary Habits

Besides the importance of drinking patterns, a growing body of evidence supports dietary habits may affect the risk of developing oral cancer and, particularly, high fruit and vegetable consumption has been associated with a reduced risk of cancer [32]. The presence of hundreds of phytochemicals, also called nutraceuticals, in plant-derived foods, may exert health-promoting effects [30]. Nutritional habits including wine consumption, such as those belonging to the Mediterranean diet, may promote health benefits possibly due the synergistic effects between wine phytochemicals and nutraceuticals of fruit and vegetable [33,34]. Evidence correlated the adherence to this dietary style to a lower risk of overall cancers, including oral and pharyngeal ones [35,36,37]. Studies showed that the incidence and mortality rates for cancers of lip, oral cavity and pharynx decreased in Mediterranean countries, such as Italy, where red wine, in the context of a healthy diet, is commonly consumed [1,2] (Table 1). Accordingly, the “Mediterranean drinking pattern” has been emphasized, supporting as the relation between alcohol consumption and all-cause mortality follows a J-shaped curve, where the low and regular wine intake seems not to be harmful for health, possibly by virtue of phytochemical components [38].

## 3. Beneficial and Harmful Components of Wine

The overall effects of wine drinking on health can be ascribed to the opposite role of phytochemicals and ethanol metabolites.

### 3.1. Wine Phytochemicals

Wine and grape polyphenols are phytochemicals belonging to phenylpropanoids, a large group of plant secondary metabolites derived from phenylalanine. They are divided into flavonoids (including anthocyanins), stilbenes (e.g., resveratrol) and proanthocyanidins (also called condensed tannins), and possess different molecular and biochemical targets both in healthy and damaged cells.

The health-promoting effects of polyphenols can be ascribed to a number of *in vitro* activities, including antioxidant, antimicrobial, antiviral, anti-inflammatory and anticancer activities [28]. On the other hand, some concerns can arise from the heterogeneous content of polyphenols in food. Indeed, a recent study on wine grapes found a high variation in the phytochemical content of different cultivars [39], thus suggesting that phytochemical level would differ in wines produced in different geographical areas.

Additional concerns are associated to preclinical evidences supporting many phytochemicals exert beneficial antioxidant effects when assumed in low to moderate doses, but can become prooxidant in case of high intake. Prooxidant activity can be beneficial against cancer cells, inducing apoptosis by production of toxic reactive oxygen species (ROS), but can become harmful if the oxidative stress damages healthy cells [28]. Thus far, there is no clinical study on the association of wine polyphenols and the risk of oral cancer, although a handful of studies, investigating phytochemicals and oral carcinogenesis, have been carried out on oral cancer cell lines and animal models.

The health benefits of resveratrol, the most evocative red wine polyphenol, have been ascribed to its antioxidant, anti-inflammatory and anticancer properties, as well as to its effects on sirtuins, involved in the epigenetic regulation of ageing [20]. Preclinical studies on anticancer activity of resveratrol date back to almost two decades ago, when, in their seminal paper, Pezzuto and colleagues demonstrated its chemopreventive potential *in vivo* [40]. Few years later, Elattar and Virji reported similar results on oral cancer [41], then confirmed by other studies: In particular, the antiproliferative activity of red wine and its major components, resveratrol and quercetin, reduced the growth and DNA synthesis in human OSCC cells [42].

In addition, new bioactive molecules have been recently discovered in grapes and wine, *i.e.*, melatonin and phytosterols [20]. The former is an indoelamine traditionally considered a vertebrate neurohormone, while phytosterols include three main compounds, *i.e.*, β-sitosterol, stigmasterol and campesterol, effective hypocholesterolizing agents.

All these phytochemicals and, possibly, others still unknown, may synergistically maximize the healthy properties of wine. Interestingly, anticancer activity of grape juice, *i.e.*, an assortment of wine phytochemicals without ethanol, was demonstrated by *in vitro* and *in vivo* models of tongue carcinogenesis [43]. However, the effective concentrations of these wine compounds are reported, in preclinical studies, at sub- to low-micromolar levels, which are at least one order of magnitude higher than those normally measured in human plasma after the oral intake of plant-derived foods, *i.e.*, tens to hundreds nanomolar [44,45]. Consistently, clinical data on pharmacokinetics support the hypothesis that these dosages are difficulty achievable following a light to moderate drinking pattern: more than 100 glasses of wine/day should be required to reach effective blood concentrations of resveratrol [46]. This is more problematic even taking into account the inter-individual differences in polyphenol oral bioavailability.

As a result, preclinical evidences regarding pharmacological activities of wine phytochemicals have not been fully substantiated by clinical trials and, besides the low oral availability of polyphenols, further factors, mainly ethanol and its metabolites, can counteract or nullify the protective effects of wine phytochemicals in human. Besides the methodological problems of in human studies, which, for instance, do not provide data on the active forms of phytochemicals, discrepancies between epidemiological and pre-clinical studies may also arise from the basic difference between the *in vivo* and *in vitro* action mechanisms of polyphenols [28]. As an example, the human metabolism of phytochemicals, involving their biotransformation by phase I and II enzymes, can alter their redox potential; this is confirmed by the lower antioxidant capacity of conjugates and metabolites over their aglycones [28].

### 3.2. Ethanol Metabolites

The most commonly recognized carcinogenic compounds in alcoholic beverages are ethanol and its main metabolite, acetaldehyde, classified as Group 1 carcinogens (“carcinogenic to humans”) by IARC [17,47]. The IARC monograph reported their causal association with several cancers, including oral carcinoma [17]. A further wine component, which may play a detrimental role, has been identified in urethane (ethyl carbamate), classified as “probably carcinogenic to humans”, activating carcinogenetic pathways different than those of ethanol [17].

Even at low dosages, ethanol may enhance the risk of cancer in those sites, such as oral cavity, in direct contact with it, via acetaldehyde damage to DNA and production of ROS, thus decreasing the cell endogenous antioxidant pool [9]. Feller *et al.* supported the role of ethanol and acetaldehyde as carcinogens by analyzing preclinical studies on its prooxidant activity in saliva [48], although a further interventional study on healthy volunteers did not find significant outcomes of a glass of red wine on salivary antiradical capacity [49]. In addition, ethanol also activates other procarcinogens, as those contained in tobacco smoking, industrial effluents and environmental pollutants, increasing the permeability of mucosa epithelial layers, enhancing the solubilisation of xenobiotics and their entrance throughout oral mucosa, and activating the mono-oxygenase enzyme cytochrome P450 2E1 (CYP2E1) involved in xenobiotic biotransformation [17,50].

Genetic factors, related to reduced ethanol and acetaldehyde metabolism, further increase the risk of oral cancer. They mainly involve genes associated to the enzyme alcohol dehydrogenase (ADH), which oxidizes ethanol to acetaldehyde, which is further detoxified by the aldehyde dehydrogenase 2 (ALDH2) to acetate, a less toxic compound. Genetic variants of these enzymes result in their low activity and decrease the clearance of ethanol and acetaldehyde from tissues. ALDH2 polymorphism, typically observable in East Asian subjects, correlates to high levels of acetaldehyde, in turn related to high risk of oral cancer [51,52,53]. In addition, endogenous salivary ADH is not the only responsible for the acetaldehyde production in the oral cavity: bacterial ADHs contribute to raise the salivary acetaldehyde levels and, consequently, may contribute to the epithelial cell proliferation [54,55] and DNA adduct formation [56]. Therefore, oral bacteria and, possibly, oral hygiene appear to play an additional pivotal role in ethanol metabolism.

## 4. Wine Drinking and Oral Cancer Risk: The in Human Evidences

In the attempt to identify a “cut-off dose” of ethanol for an increased risk of oral cancer, research revealed, in the past, a major drawback related to a not uniform way of reporting the total volume of individual ethanol intake. To overcome this issue, in recent years, standard units, namely “standard drinks” as g of ethanol/drink, have been proposed at supranational level to obtain a predictable relative risk, even if they are still not homogeneously accepted worldwide [57,58] (Table 2).

**Table 2:** Definition of “standard drink” in different countries. Source: International Center for Alcohol Policies [57].

Standard Unit (in g of ethanol)	Country
8.0	United Kingdom
9.9	Netherlands
10.0	Australia, France, Hungary, Ireland, New Zealand, Poland, Spain
11.0	Finland
12.0	Denmark, Italy, South Africa
13.6	Canada
14.0	Portugal, United States

On what concerns the type of beverage, wine-related data can be extrapolated from human trials on alcoholic beverage intake, particularly from systematic reviews, observational studies and biokinetic clinical trials on wine phytochemicals. The following paragraphs describe retrieved studies, also summarized in Table 3.

**Table 3:** Oral cancer risk and alcohol drinking.

Reference	Type of Article	Wine *	Oral Cancer Risk
[11]	Meta-analysis	No	Oral and pharyngeal cancer RR ^1^ = 5.13 for heavy drinkers.
[59]	Meta-analysis	No	Upper aero-digestive tract cancer: RR = 2.97 (but RR = 2.24 only considering oral cavity, pharynx, and larynx); alcohol intake increasing of 10 g/day equivalent to increasing RR of 1.09.
[26]	Meta-analysis	**Yes**	Wine-specific RR = 2.1 for any drinking pattern; RR = 4.92 for heavy drinking (≥4 drinks/day).
[27]	Meta-analysis	No	RR = 1.05 for light drinkers.
[13]	Meta-analysis	No	25% Alcohol-attributable cases for upper aero-digestive tract cancer.
[12]	Meta-analysis	No	30% Alcohol-attributable cases for oral cavity and pharynx cancer with an increased risk related to 1 g/day of ethanol of 0.0185%.
[26]	Meta-analysis	No	RR = 1.3 for 10 g/day, 3.2 for 50 g/day, 8.6 for 100 g/day and 13.0 for 125 g/day of ethanol.
[60]	Meta-analysis	No	RR = 1.1 for light drinkers; RR = 4.6 for heavy drinkers.
[8]	Meta-analysis	No	Oral cancer RR = 1.65 for 1–39 g/day of ethanol in men; RR = 1.43 for 1–19 g/day in women.
[61]	Meta-analysis	No	Oral cancer RRs = 1.86, 3.11 and 6.45 for ethanol intake of 25, 50 and 100 g/day, respectively.
[62]	Meta-analysis	No	Oral cavity and pharynx cancers RRs = 1.75 (25 g/day of ethanol), 2.85 (50 g/day), 6.01 (100 g/day).
[63]	Meta-analysis	No	Oral cancer RRs = 2.2, 4.2 and 10.7 for ethanol intake of 25, 50 and 100 g/day, respectively.
[64]	Cohort study (Netherlands)	No	Oral cavity cancer: RRs = 1.25, 1.91, 3.88, 6.39 for 0–5, 5–15, 15–30, ≥30 g/day of ethanol; for regular consumers: RRs = 1.65, 1.68, 3.20, 7.50 in the same order; wine specific: RR = 1.07, 1.31, 0.93, for intake of 0–1, 1–2, ≥2 glasses/day.
[30]	Cohort study (Korea)	No	Daily binge drinkers *versus* non binge-drinkers: oropharyngeal cancer mortality HR ^2^ = 4.82; adjusting for the volume of alcohol intake and frequency of binge, HR = 4.90.
[65]	Case-control (Brazil)	No	Drinking was not independently associated with oral cancer; drinking status: ever drinker OR ^3^ = 4.21, level-1 drinker (≤862 g/year) OR = 1.68, level-2 drinker (>862 g/years) OR = 6.73; drinking and smoking status: never smoker and ever drinker OR = 0.58, ever smoker and ever drinker OR = 5.85.
[4]	Case-control ICARE study (France)	No	Population-attributable risk of oral cavity cancer 7.3% for alcohol drinking.
[7]	Case-control ARCAGE multi-centre study (10 European countries)	No	Oral cancer OR = 1.04 related to alcohol alone; OR = 7.06 related to alcohol/smoking joint effect.
[66]	Case-control (Turkey)	**Yes**	Oral cancer OR = 0.549 for red wine intake.
[28]	Ecological study (Europe, North America, Oceania and Far Eastern Asia)	**Yes**	Male age-standardised mortality rate for oral cancer: significantly increasing for every litre of pure ethanol (0.15 per 100,000 subjects) and spirits (0.26 per 100,000 subjects), but non-significant effects for beer and wine.
[67]	Case-control (4 European countries, Cuba, Canada, India, Sudan and Australia)	**Yes**	ORs = 2.86 for ever drinker, 2.12 for ex-drinkers, 3.46 for current drinkers; type of drink: only beer OR = 1.16, only wine and beer OR = 1.96, only wine OR = 2.71, spirits with or without wine or beer OR = 7.28; drinking amount (independently from type of beverage): for 1 drink/day OR = 2.00, for 2 drinks/day OR = 3.74, for 3–4 drink/day, OR = 6.22, for 5–6 drink/day OR = 10.58, for 7–10 drink/day OR = 10.29.
[68]	Case-control (Italy and Switzerland)	**Yes**	Wine, OR = 1.0 for 1–2 drinks/day, 2.2 for ≥3 drinks/day.
[69]	Case-control (Southern Greece)	**Yes**	OR = 1.7 for moderate drinkers (1–28 drinks/week); ORs = 0.8 and 1.1 only considering wine drinkers of 1 drinks/week and ≥14 drinks/week, respectively.
[70]	Case-control (Spain)	**Yes**	OR = 1.89 for 1–50 g/day of alcohol; OR = 5.3 in wine drinkers exceeding 50 g/day of ethanol (*i.e.*, 4 glasses per day).
[6]	Cohort study (Denmark)	**Yes**	RR = 3.0 for drinkers of 7–21 beers or spirits/week, but not wine, compared with non-drinkers; RR = 0.5 for subjects with the same total alcohol intake, but with wine (>30% of their intake); RR = 5.2 for drinkers of >21 beers and spirits/week but not wine, RR = 1.7 for subjects with the same total alcohol intake, but including wine.
[71]	Case-control (Japan)	No	OR = 3.6, 4.5 and 4.8 for sake, beer and hard liquor drinkers, respectively.
[72]	Case-control (Italy)	**Yes**	OR = 11.2, 9.9 and 4.1 among heavy drinkers (≥84 drinks/week) of wine only, wine and spirits and combination wine-spirits-beer.
[73]	Case-control (North Italy)	**Yes**	OR = 4.9 for heavy wine drinkers (≥56 glasses/week, *i.e.*, about 1 litre/day), rising to 8.5 for drinkers of ≥84 glasses/week.
[49]	Salivary biokinetics (Caucasoid healthy subjects)	**Yes**	Acute intake of 125 mL of red wine: no effect on anti-radical salivary capacity, but administration of red wine polyphenol capsules improved the salivary antioxidant status.
[52]	Salivary and blood biokinetics (Asian healthy subjects)	**Yes**	Acute intake of 0.6 g ethanol/kg body weight in the form of 13% ethanol Calvados, 13% ethanol *shochu*, 13% ethanol red wine and 5% ethanol beer.
[74]	Salivary and blood biokinetics (Asian heavy drinkers-alcoholics)	No	Patients with homozygous alcohol dehydrogenase-1B (ADH1B*1/*1), who drunk the day before, were associated with higher levels of ethanol persisting in the blood for longer periods and had higher salivary acetaldehyde levels, correlating to oral bacteria and yeast counts; no effect of inactive heterozygous aldehyde dehydrogenase-2 (ALDH2*1/*2) was observed on ethanol lingering the next morning.
[75]	Salivary and blood biokinetics (Asian healthy subjects)	No	Acute intake of 0.4 g ethanol/kg body weight in a standardized 10% (*v*/*v*) solution of absolute ethanol in orange juice (with and without previous ingestion of 4-methylpyrazole, an inhibitor of human ADH): a high salivary production of acetaldehyde by oral microflora alcohol dehydrogenase was observed.
[55]	Salivary and blood biokinetics (Asian and Caucasoid healthy subjects)	No	Acute intake of 0.5 g ethanol/kg body weight in a standardized 10% (*v*/*v*) solution of absolute ethanol in orange juice: Aldehyde dehydrogenase-2 (ALDH2) deficient subjects had 2–3 times higher salivary acetaldehyde levels than ones with normal ALDH2, after 240 min; salivary acetaldehyde originated from oral microflora and parotid gland ethanol metabolism.
[54]	Salivary and blood biokinetics/cohort study (Finland, healthy subjects, dental and oral cancer patients, heavy drinkers—alcoholics)	No	Smoking and heavy alcohol intake increased salivary acetaldehyde; considering alcoholic status, levels of acetaldehyde were: teetotalers 111 μmol/L, moderate alcohol consumption 104 μmol/L, heavy drinkers 172 μmol/L.
[76]	Salivary and blood biokinetics (Caucasoid healthy subjects)	No	Acute intake of 0.5 g ethanol/kg body weight in a standardized 10% (*v*/*v*) solution of absolute ethanol in orange juice; salivary acetaldehyde was associated with oral microflora: it peaked within 40 min after ethanol ingestion and decreased after a 3-day use of antiseptic mouthwash (chlorhexidine).

* Wine-specific analysis of data for oral cancer risk; ^1^ RR, relative risk; ^2^ HR, hazard ratio; ^3^ OR, odd ratio.

### 4.1. Systematic Reviews

Recent meta-analyses have causally correlated oral cavity cancer to alcohol consumption over recommended doses, reporting the 25% and 44% of cancer cases attributable to ethanol in woman and man, respectively, although these data are only based on case-control studies [61,63]. In 1999, the relative risk (RR) of oral cancer for subjects drinking 25 g of ethanol/day (two drinks/day) was 2.2 and followed a dose-dependent increase [63], also confirmed by recent studies [59,76]. In 2004, when more studies became available, the RR changed to 1.86 for the same ethanol consumption [61], decreased to 1.2 for the intake of ≤1 drink/day [62], whereas raised to 5.2 for heavy alcohol drinkers (≥4 drinks/day) [77]. Consistently, in 2006, the RRs of oral cancer corresponded to 1.65 in men with an overall intake of 1–39 g of ethanol/day, and to 1.43 in women with the same ethanol intake [9].

Considering data only on light and regular alcohol consumption, a recent meta-analysis suggested a RR of oral cancer corresponding to 1.05, independently from the type of beverage [10], even weaker in never/non-current smokers [26,78]. Further estimations of RRs for light drinkers, in comparison to abstainers or occasional alcohol consumers, were 1.1 for oral cancers, 1.2 for pharyngeal cancers [26] and 1.26 for upper aero-digestive tract cancers [59,76]. As regards type of beverage, overall RRs for any drinking pattern and independently from smoking status, corresponded to 2.1, 2.4, and 2.3 for wine-, beer- and spirits-only intakes, respectively [26]. Heavy drinkers showed RRs equal to 4.9, 4.2, and 5.2, following the same order of beverages, whereas results on light to moderate drinking were not recorded because of limited data availability [26].

### 4.2. Observational Studies

The most prevalent alcoholic beverage in a given population is commonly the most heavily consumed one, in turn most likely to be associated with increased oral cancer risk [6,13,79]. For instance, in the Italian population, heavy drinking of wine, the predominant beverage consumed, appeared to produce the highest threat when compared with similar intake of beer and spirit [72,73,79]. As expected, the risk increased in a dose-dependent trend [64,67,68,70,73], corroborating the importance of the drinking pattern. In 2014, a Korean study associating heavy binge drinking and oro-pharynx cancer mortality showed a hazard ratio (HR) of 4.90, whereas the latter was 1.50 for non-binge drinkers [30]. Petty and Scully reported mortality rate for oral cancer significantly increased for every litre of pure ethanol (0.15 per 100,000 subjects) and spirits (0.26 per 100,000 subjects), though without significant effects for beer and wine [28]. Similarly, a case-control study carried out in Italy and Switzerland demonstrated that, compared to abstainers or light drinkers, overall odds ratio (OR) for oral cancer was 2.1 for three to four drinks/day, decreasing to 1.0 for one to two drinks/day of wine [68]. These data are consistent with studies performed on Turkish [66], Greek [69] and Japanese populations [71], reporting higher risk associated to liquor consumers than wine drinkers. A Spanish case-control study also reported increased risk of oral cavity or oropharynx cancers among individuals drinking 1 drink per day (overall OR = 2.0), which was higher for spirit consumers than wine drinkers (OR of 7.2 and 2.7, respectively) [67]. Finally, the Netherlands Cohort Study found a RR for oral cancer of 1.68 for “stable” drinkers of 5–15 g ethanol/day (people who did not change the habit in the last five years); interestingly, the only wine consumption of 0–1 or 1–2 glasses/day gave RRs of 1.07 and 1.31, respectively [64].

Considering the combined effect of tobacco and ethanol, strong evidence correlated high levels of alcoholic beverage consumption to heavy smoking habit [73]. This dose-dependent and synergistic effect was confirmed in the large French ICARE study, showing the 81% of oral cancer risk attributable to tobacco and ethanol exposures, with smoking responsible for the higher risk [32,80]. These findings are consistent with the European ARCAGE case-control study: oral cancer OR was 1.04 related to alcohol alone and increased to 7.06 in presence of combined ethanol and tobacco use [7,70,81].

Tobacco smoking is the main risk factor for the majority of SCC of the oral cavity. When studying the role of alcohol in oral carcinogenesis, it is necessary to adjust for tobacco use and, more importantly, to conduct the study on never smokers. As reported above, many epidemiologic studies focused on the association between alcohol intake and oral cancer risk, and most have adjusted for tobacco use; conversely, a number of studies investigated the influence of alcohol on oral cancer risk among never users of tobacco. In the study from INHANCE (International Head and Neck Cancer Epidemiology Consortium), the association between ethanol intake (from light to heavy: 0, <1, 1–2, 3–4, >5 drinks/day) and the risk of head and neck (oral cavity, oropharynx and larynx) cancers among never users of tobacco was examined [32,80]. However, some observational studies, which do not take into account a number of confounding factors, need to be carefully interpreted, in order to avoid the potential overestimation of smoking and drinking independent roles. After adjustment for age, sex and instruction in multivariable analyses, drinking seems not to be an independent risk factor for oral cancer [65]. To further complicate the interpretation of the results, important publication biases should be considered: they include drinker’s under-reporting of alcohol consumption [82] or frequent incorrect categorization of former drinkers into non-drinker group [10].

### 4.3. Biokinetics Studies

Low oral bioavailability and poor systemic delivery to target tissues and organs represent the major drawbacks associated with the clinical failure of many promising phytochemicals [83]. Pharmacokinetic studies on resveratrol, quercetin and anthocyanidines were carried out in healthy volunteers after grape juice and moderate red wine consumptions: results showed that glycosides, mainly glucose conjugates, are absorbed to a lesser extent than the corresponding aglycones [82,84]. Within the oral cavity, hydrolysis of anthocyanidines, quercetin and genistein glucosides to their aglycones by salivary, bacterial and cellular β-glucosidases was demonstrated, with a remarkable inter-individual variability [42]. Similarly to small intestine, phase II and efflux transporting enzymes are also present in human oral mucosa, as well as glucuronidated anthocyanin conjugates, can be detected in saliva [42]. Oral cavity tissues are in direct contact with wine and related bioactive phytochemicals. Hence, the levels of salivary polyphenols peaked soon after red wine intake in healthy volunteers and appeared to derive mainly from a reservoir adhering to oral mucosa, rather than from systemic absorption [85,86].

Besides studies on polyphenol biokinetics in oral fluids, many investigations focused on the analysis of salivary acetaldehyde. Apart from the systemic ethanol metabolism, a 30 second rinsing of the oral cavity with different alcoholic beverages, including wine, increased the salivary acetaldehyde levels over the threshold previously shown to be carcinogenic by *in vitro* models [85]. However, a cross-over randomized clinical trial on Japanese volunteers, focusing on wine intake, reported that the salivary concentration of acetaldehyde was lower soon after drinking a glass of 13% wine in comparison to other alcoholic beverages [52]. A further element affecting salivary acetaldehyde is the smoking habit, since smokers showed higher level of this compound, probably related to oral bacteria metabolism [54].

Finally, high-risk genotypes of salivary alcohol and aldehyde dehydrogenases, *i.e.*, ADH1B and ALDH2, respectively, commonly reported in certain Asian populations, significantly affected acetaldehyde levels in saliva [55,74,81]. Thus, the high salivary acetaldehyde production in these drinkers represents a plausible explanation for the enhanced cancer risk observed, partly attributable to prolonged ethanol and acetaldehyde exposures because of the less-active ADH1B and ALDH2 enzymes, as well as to oral microflora over-growth [55,75,86]. Noteworthy, many epidemiological studies showed the ALDH2 deficiency gene (ALDH2*2 allele) to be associated with markedly increased risk of digestive tract cancers, including oro-pharyngeal cancers, even after a moderate dose of alcohol intake [55,86]. In particular, it has been suggested that individuals with normal ALDH2 need to drink about two to three times more alcohol per occasion to achieve the same salivary acetaldehyde levels of subjects with the deficient isoenzyme [55,86].

## 5. Conclusions

Drinking pattern, dietary habit and type of population under investigation can affect the cancer risk. Although little evidence exists specifically focusing on light wine intake, a low, but present, risk of oral cavity cancer can be observed.

Phytochemicals in wine seem not to have any influence on cancer risk, possibly because they are present at low levels and poorly absorbed, reaching in human levels much below the effective concentrations assessed in preclinical studies. Therefore, currently, there is clearly not a causal correlation between phytochemicals in wine and health outcomes, as well as it appears premature to assume that phytochemical and ethanol, in this beverage, play a balancing role on oral carcinogenesis, with the former being protective and the latter being harmful.

Noteworthy, when ethanol consumption is coupled to smoking habit or to high-risk dehydrogenase genotypes, the oral cancer risk dramatically increases. The clinician should always assess the patient-specific risk, considering the additive/synergistic behaviors, including drinking pattern, dietary habits and smoking status together.
